# Defining the learning curve for endotracheal intubation in the emergency department

**DOI:** 10.1038/s41598-022-19337-8

**Published:** 2022-09-01

**Authors:** Gun Tak Lee, Jong Eun Park, Sook-young Woo, Tae Gun Shin, Daun Jeong, Taerim Kim, Se Uk Lee, Hee Yoon, Sung Yeon Hwang

**Affiliations:** 1 Department of Emergency Medicine, Samsung Medical Center, Sungkyunkwan University School of Medicine, 81 Irwon-Ro, Gangnam-Gu, Seoul, 06351 Korea; 2 Department of Emergency Medicine, College of Medicine, Kangwon National University, Chuncheon, Gangwon-Do Korea; 3 Biomedical Statistics Center, Data Science Research Institute, Samsung Medical Center, Samsung Medical Center, Seoul, Korea

**Keywords:** Medical research, Outcomes research

## Abstract

To determine the minimum number of endotracheal intubation (ETI) attempts necessary for a novice emergency medicine (EM) trainee to become proficient with this procedure. This single-center study retrospectively analyzed data obtained from the institutional airway registry during the period from April 2014 to March 2021. All ETI attempts made by EM trainees starting their residency programs between 2014 and 2018 were evaluated. We used a first attempt success (FAS) rate of 85% as a proxy for ETI proficiency. Generalized linear mixed models were used to evaluate the association between FAS and cumulative ETI experience. The number of ETI attempts required to achieve an FAS rate of ≥ 85% was estimated using the regression coefficients obtained from the model. The study period yielded 2077 ETI cases from a total of 1979 patients. The FAS rate was 78.6% (n = 1632/2077). After adjusting for confounding factors, the cumulative number of ETI cases was associated with increased FAS (adjusted odds ratio, 1.010 per additional ETI case; 95% confidence interval 1.006–1.013; p < 0.001). A minimum of 119 ETI cases were required to establish a ≥ 85% likelihood of FAS. At least 119 ETI cases were required for EM trainees to achieve an FAS rate of ≥ 85% in the emergency department.

## Introduction

Endotracheal intubation (ETI) is a frequently used life-saving procedure for critically ill patients in emergency departments (EDs). Thus, ETI is a core aspect of the emergency medicine (EM) training curriculum. However, it is a complex psychomotor skill that carries a significant risk of procedure-related complications. Therefore, substantial practice is required to become proficient with this technique.

The Accreditation Council of Graduate Medical Education (ACGME) classified ETI as a key index procedure essential for the independent practice of EM^1^. As is the case with any manual technique, ETI skill has a learning curve. Setting goals for the minimum experience required to be proficient with this technique is critical when planning an appropriate EM training program. The ACGME requires EM residents to perform at least 35 ETIs during their training^1^. Although some studies have been conducted on the learning curve of ETI for EM trainees, there is insufficient data to support this minimum experience requirement^2,3^. Additionally, the learning curves for ETI differed between studies based on the statistical methods, study population, devices used, study setting, and the proficiency definition used^2–9^.

In the field of EM, a universally accepted definition of proficiency for psychomotor skills, such as ETI, is lacking. In prior studies that examined the learning curve for ETI, researchers employed different criteria to describe proficiency^2,3,10^. A common indicator of one's proficiency for ETI is the first attempt success (FAS). In current practice, FAS at emergency ETI is prioritized, as several studies have shown that an increased number of ETI attempts is associated with an increased risk of adverse outcomes. In a previous systematic review and meta-analysis that included a total of 42,081 ETIs from 83 institutions in 10 countries, the FAS in the ED was 84.1% (95% confidence interval [CI] 80.1–87.4])^11^. Given the findings of the previous study and the relevance of FAS rates to patient safety, it may be reasonable to set the proficiency criterion for EM physicians at ≥ 85%.

Our study aimed to determine the number of ETI cases required for a novice EM trainee to achieve a proficiency level, defined as achieving an FAS rate of ≥ 85%.

## Methods

### Institutional review board statement

The study was conducted in accordance with the Declaration of Helsinki. The Samsung Medical Center Institutional Review Board approved this study and waived the requirement for informed consent due to the anonymity and retrospective nature of the study.

### Study design and setting

This single-center retrospective study was conducted at the ED of the Samsung Medical Center (a tertiary referral hospital in an urban area, with an annual ED census of > 70,000 patients). The study data were retrieved from the institutional airway registry between April 2014 and March 2021, and all the ETI attempts that the EM trainees who began their residency program between 2014 and 2018 made throughout their entire training period was evaluated.

This ED is affiliated with a residency training program in EM and generally accepts four or five trainees. Thirty-four residents began their first year of EM residency during the study period. In South Korea, a newly licensed medical doctor who has completed medical school is required to undergo a one-year internship before beginning residency training in each specialty field, including EM. During the internship course, they receive basic medical training while rotating across several departments, typically for a month. As a high-level technique, ETI is rarely assigned to interns, and only a few departments, such as the anesthesia department, give restricted opportunities for ETI. Thus, EM trainees who have completed their internships usually have a very limited experience with ETI when they enter the residency program in South Korea. The residency program in EM requires 4 years of training, which begins in March each year.

ETI is performed on approximately 300–400 adult patients annually, and EM physicians perform the majority of ETIs in the ED. EM trainees commonly engage in ETIs to gain proficiency with the technique. Trainees should be required to perform ETIs under the supervision of EM faculty. EM trainees are generally allowed up to two ETI attempts per patient to minimize the risk of compromising patient safety. However, this limit could be waived depending on the supervisor's judgment or patient's condition. If the trainee cannot successfully complete the ETI, the senior resident or faculty member takes responsibility for the laryngoscope. Adult patients who underwent at least one ETI attempt by an EM trainee who began their residency program between 2014 and 2018 were included in this study. ETIs that were performed exclusively by EM specialists, EM residents who began their first year of residency prior to 2014 or after 2018, or by non-EM physicians were excluded from the study.

### Data collection and outcome measures

Every ETI performed on adult patients in the ED during the study period was recorded with detailed patient and procedural data in the institutional airway registry, regardless of the time of day. No ETI attempt in the ED was omitted during the study period. The entire procedure of each ETI was monitored and recorded in real-time by an independent staff member using a standardized data entry format to minimize recall and reporting bias^12^. The intubator and faculty members reviewed and completed the data after the procedure. The faculty responsible for the registry regularly monitored the data for quality control.

We extracted the following data from our institutional registry and electronic medical records: baseline characteristics of the patients, including age, sex, and body mass index (BMI); the presence of difficult airway characteristics; reason for ETI; intubating devices (conventional Macintosh-type direct laryngoscope [DL], C-MAC video laryngoscope [C-MAC VL, Karl Storz Endoskope, Tuttlingen, Germany], and Pentax Airwayscope [PAS]); and number of ETI attempts.

An ETI attempt was defined as insertion of the laryngoscope blade into the mouth, regardless of successful endotracheal tube placement into the trachea. Changing operators without removing the intubating device or endotracheal tube from the patient's mouth was also counted as an attempt. The monitoring staff strictly documented the number of attempts, because even a simple manipulation by the blade could harm the patients. Successful ETI was defined as proper endotracheal tube placement confirmed by various methods, such as ultrasonography, end-tidal carbon dioxide measurement, and auscultation. FAS was defined as successful ETI on the first attempt. An ETI case was defined as an ETI attempt by an individual trainee on a patient. In the case of multiple attempts on a single patient by two or more trainees, if the first trainee failed but the second trainee successfully intubated the same patient, it was counted as one case for each trainee. We classified the patient’s BMI into the following four categories according to the categories specified by the World Health Organization: underweight (< 18.5 kg/m^2^), normal weight (18.5–24.9 kg/m^2^), preobese (25.0–29.9 kg/m^2^), and obese (≥ 30.0 kg/m^2^)^13^.

Anticipated difficult airway was determined by an intubator, if one or more of the following characteristics were observed in the patient during the evaluation prior to ETI: external appearance such as a short neck, facial trauma, small mandible, or obesity; Mallampati class ≥ 3; airway obstruction, including airway edema or tracheal stenosis; cervical immobilization; and limited mouth opening of < 3 cm. Due to the lack of patient participation and time constraints, it might be challenging to apply complex criteria with precise measurements in an emergency situation. Thus, although some criteria were vulnerable to subjectivity, we employed a list of difficult airway features that were feasible for the intubator to assess prior to ETI using a quick and straightforward evaluation tool. The device was selected at the discretion of the intubator or the on-duty EM faculty.

We used an FAS rate of ≥ 85% as a proxy for ETI proficiency. We also evaluated the minimum number of ETI attempts required to achieve an FAS of over 80% and 90%, respectively, and a success rate of ≥ 90% within two ETI attempts.

### Data analysis

Continuous data were presented as medians with interquartile ranges (IQR, 1st quartile–3rd quartile) and categorical data as frequencies with percentages. The variables were compared using either the Mann–Whitney *U* test or the chi-square test, as applicable. For univariable and multivariable analyses, a generalized linear mixed model (GLMM) with a random intercept and a logit link function was used to analyze the association between FAS and cumulative ETI cases and to establish the equation for predicted probability of FAS based on the cumulative number of ETI cases.

The coefficient and intercept of the cumulative number of ETI cases for FAS were estimated using GLMMs with a random intercept, and they were used to predict the probability of FAS. The specific predictor value was multiplied by the coefficient of the model corresponding to the cumulative number of ETI cases and then added to the intercept value of the model. The following formula was used to determine the predicted FAS probability:

$$\mathrm{Predicted} \, \mathrm{ probability } \, \mathrm{ of } \, \mathrm{ FAS }=\frac{{e}^{0.95321 + 0.006586*\mathrm{cumulative} \, \mathrm{  ETI } \, \mathrm{ case}}}{1+ {e}^{0.95321 + 0.006586*\mathrm{cumulative} \, \mathrm{  ETI } \, \mathrm{ case}}}$$

Using this prediction equation, the number of cumulative ETI cases in which the FAS rate is at least 85% was determined inversely.

Variables with a p-value < 0.1 from univariable analyses were selected for the multivariable model. The following variables were included in the multivariable model: cumulative number of ETI attempts, device used for ETI (DL vs. VL), indication for ETI (cardiac arrest vs. non-cardiac arrest), and difficult airway (difficult vs. non-difficult airway). The results were presented as adjusted odds ratios (aOR) with 95% CIs. A statistical significance was defined as a p-value < 0.05. Statistical analysis was performed using R 4.1.0 (Vienna, Austria; http://www.R-project.org/).

## Results

All trainees had fewer than five previous ETI experiences, except for one who had < 10 experiences.

### Baseline characteristics

**Figure 1:**
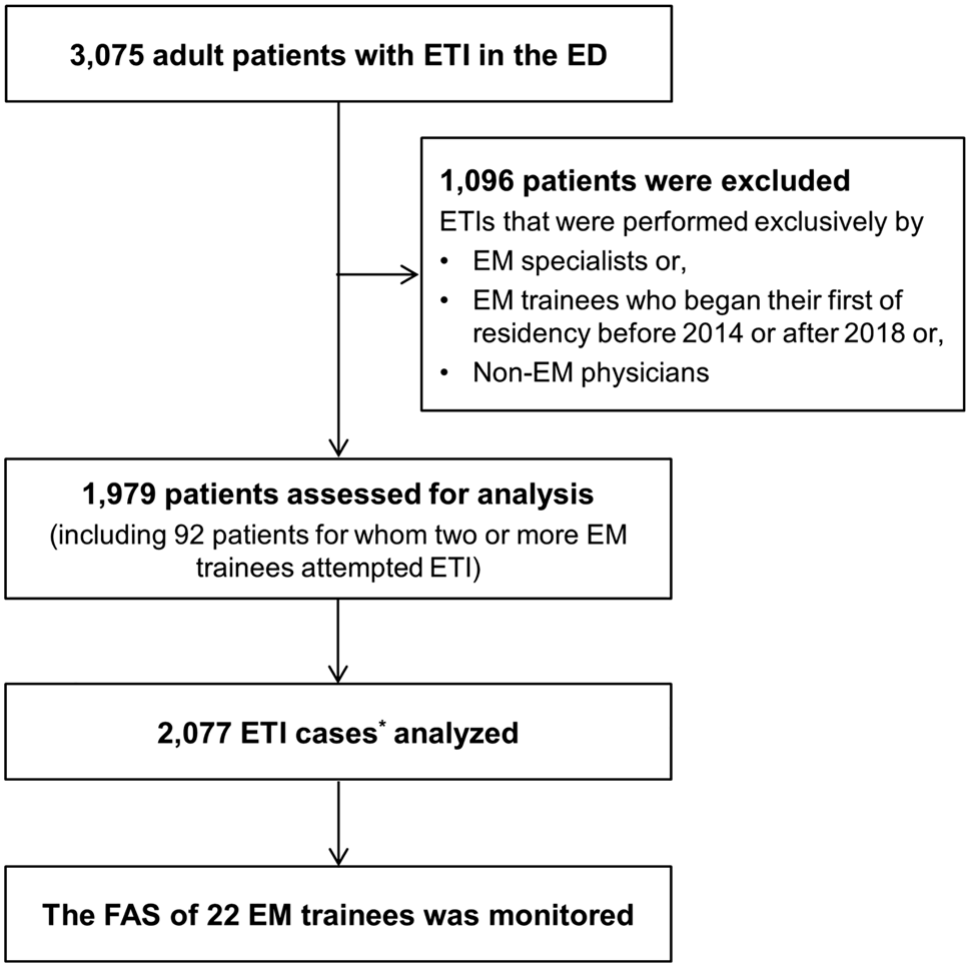
Study flowchart. *ETI* endotracheal intubation, *ED* emergency department, *EM* emergency medicine, *FAS*, first attempt success. *An ETI case was defined as an ETI attempt by an individual trainee on a patient.

Of the 3075 adult patients who underwent ETI during the research period, 1096 were excluded because they met the exclusion criteria, leaving 1979 patients for analysis. The evaluation of 1979 patients yielded a total of 2077 ETI cases (Fig. 1). The baseline characteristics of the patients are shown in Table 1. The median age was 67 years (IQR 55–77), and 62.8% of the patients were male (n = 1243/1979). A total of 25.5% (n = 504/1979) of the patients were classified as having a difficult airway. Cardiac arrest (37.2%, n = 737/1979) was the most common indication for ETI, followed by respiratory failure (30.4%, n = 602/1979) and airway protection (20.5%, n = 406/1979). The overall complication rate was 11.9% (n = 236/1979).

**Table 1:** Baseline characteristics of the patients.

	Total (N = 1979)
**Patient age, (years)**	67.0 (55.0–77.0)
**Patient sex, (male)**	1243 (62.8)
**Patient BMI, (kg/m**^**2**^**)**	22.9 (20.5–25.2)
**Patient BMI grade**	
Underweight (< 18.5 kg/m^2^)	216 (10.9)
Normal weight (18.5–24.9 kg/m^2^)	1244 (62.9)
Preobese (25.0–29.9 kg/m^2^)	448 (22.6)
Obese (≥ 30.0 kg/m^2^)	71 (3.6)
**ETI indication**	
Non-traumatic	1787 (90.3)
Cardiac arrest	666 (33.7)
Altered mental status	346 (17.5)
Respiratory distress	579 (29.3)
Shock	119 (6.0)
Other medical illness	77 (3.9)
Traumatic	192 (9.7)
Cardiac arrest	71 (3.6)
Traumatic shock	15 (0.8)
Head and neck trauma	88 (4.4)
Other trauma	18 (0.9)
**Anticipated difficult airway**	504 (25.5)
**Methods for ETI**	
Crash approach*	737 (37.2)
RSI	1041 (52.6)
Sedative only	124 (6.3)
No medication	77 (3.9)
**Surgical airway**^†^	9 (0.5)
**Complications**^‡^	
Overall	236 (11.9)
Esophageal intubation	76 (3.8)
Unrecognized esophageal intubation	1 (0.05)
Post-intubation hypotension	66 (3.3)
Post-intubation hypoxia	53 (2.7)
Post-intubation arrest	37 (1.9)
Dental injury	17 (0.9)
Agitation	12 (0.6)
Vomit	2 (0.1)
Post-intubation dysrhythmia	1 (0.05)
Pneumothorax	1 (0.05)

Data are presented as medians (interquartile ranges) or numbers (%).

*BMI* body mass index, *ETI* endotracheal intubation, *RSI* rapid sequence intubation.

*Crash approach: used for unconscious, unresponsive patients expected not to be resistant to laryngoscopy and who needed immediate airway security.

^†^Patients whose airways have been secured with a surgical technique.

^‡^Postintubation cardiac arrest was defined as cardiac arrest occurring within 30 min after ETI. Postintubation hypotension was defined as a systolic blood pressure of < 90 mm Hg at any time within the first 30 min following ETI. Postintubation hypoxemia was defined as peripheral oxygen saturation of < 80% at any point within 30 min following ETI. Cardiac arrest, hypotension, and hypoxemia existing before ETI were not considered postintubation complications.

EM trainees performed a median of 82.5 ETI cases (IQR 69–123). The ETI case-related variables are listed in Table 2 (See also Supplementary Table S1). C-MAC VL (61.3%, n = 1274/2077) was the most frequently used device in the first attempt at ETI, followed by DL (34.1%, n = 709/2077) and PAS (4.5%, n = 94/2077). Overall, the FAS rate was 78.6% (n = 1632/2077) and the success rate within two attempt was 85.4% (n = 1774/2077). The combination of devices used for the first and second ETI attempts, as well as the success rate for the second attempt, are described in Supplementary Table S1.

**Table 2:** Device used for first attempt and ETI success rate.

	Total (n = 2077)
**Device used for first attempt, n (%)**	
DL	709 (34.1)
VL	1368 (65.9)
C-MAC VL	1274 (61.3)
PAS	94 (4.5)
**First attempt success rate, % (95% CI)**	
Overall	78.6 (76.7–80.3)
Patient sex	
Male (n = 1,310)	77.8 (75.4–80.0)
Female (n = 767)	79.9 (76.9–82.7)
Patient BMI grade	
Under weight (< 18.5 kg/m^2^, n = 226)	79.2 (73.3–84.3)
Normal weight (18.5–24.9 kg/m^2^, n = 1297)	79.3 (77.0–81.5)
Preobese (25.0–29.9 kg/m^2^, n = 481)	76.9 (72.9–80.6)
Obese (≥ 30.0 kg/m^2^, n = 73)	74.0 (62.4–83.5)
**Level of intubator**	
First year (n = 303)	68.7 (64.0–73.0)
Second year (n = 649)	79.1 (76.1–81.8)
Third year (n = 584)	82.0 (78.9–84.8)
Fourth year (n = 210)	84.7 (79.4–88.7)
Fellow (n = 28)	76.5 (59.5–87.8)
**ETI indication**	
Cardiac arrest (n = 780)	76.4 (73.3–79.3)
Non-cardiac arrest (n = 1297)	79.9 (77.6–82.0)
**Anticipated difficult airway**	
Yes (n = 535)	66.2 (62.0–70.2)
No (n = 1542)	82.9 (80.9–84.7)
**Success rate within two attempts, % (95% CI)**	85.4 (83.8–86.9)

*ETI* endotracheal intubation, *DL* direct laryngoscope, *VL* video laryngoscope, *PAS* Pentax Airwayscope, *CI* confidence interval, *BMI* body mass index.

### Relationship between FAS and cumulative intubation experience

**Figure 2:**
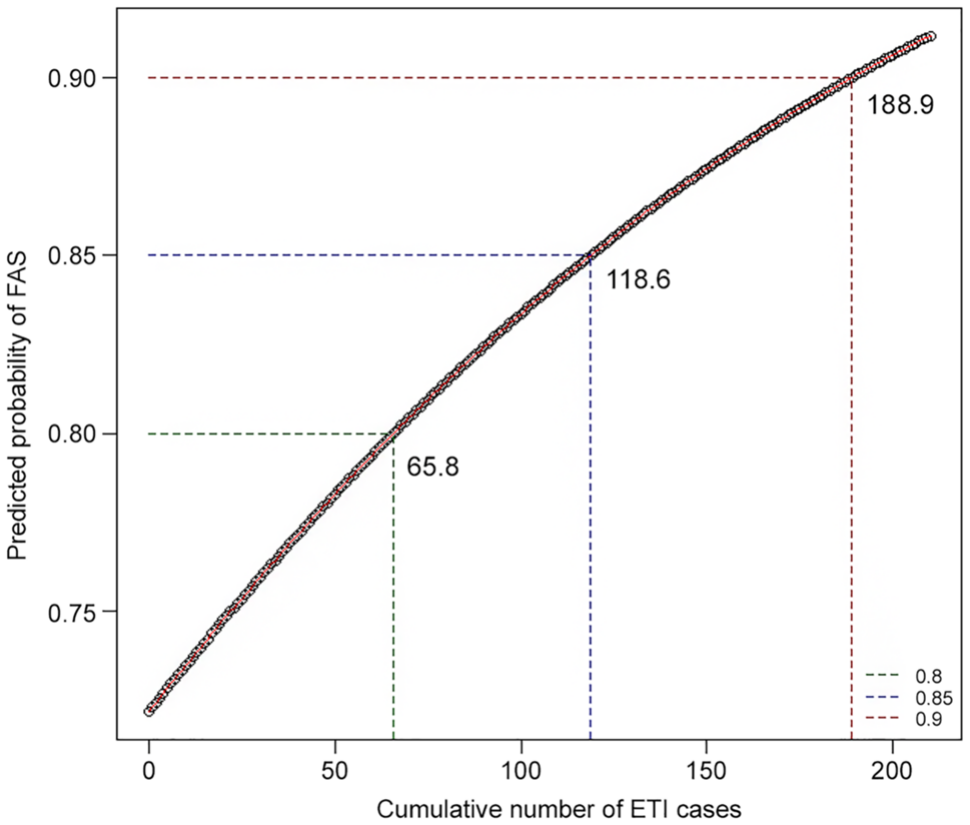
Relation between the cumulative number of ETI cases and predicted probability of first attempt success. Generalized linear mixed models with random intercept using the logit link function were used to estimate the probability of FAS according to the cumulative number of ETI cases. *FAS* first attempt success, *ETI* endotracheal intubation.

Figure 2 shows the relationship between the cumulative number of ETI cases and predicted probability of FAS, showing an increase in the predicted FAS probability with cumulative number of ETI cases. At least 119 ETI cases were needed to achieve an FAS probability of ≥ 85%. In addition, at least 66 and 189 cumulative ETI cases were required to achieve FAS probabilities of 80% and 90%, respectively. The probabilities of FAS based on intubating device, indication for ETI, and the presence of difficult airway characteristics are presented in Supplementary Fig. S1. At least 88 cumulative cases were required to achieve a success rate of > 90% within two ETI attempts (see Supplementary Fig. S2).

The results of the univariable and multivariable analyses to identify the association between FAS and cumulative intubation experience are presented in Table 3 (See also Supplementary Tables S2–S4). The cumulative number of ETI cases was associated with increased FAS (odds ratio, 1.007 per additional ETI case; 95% CI 1.007–1.010; p < 0.001). This association remained significant after adjusting for ETI device, indication for ETI, and presence of difficult airway (aOR 1.010 per additional ETI case; 95% CI 1.006–1.013; p < 0.001). Compared with DL use, VL use was significantly associated with increased FAS (aOR 1.890; 95% CI 1.506–2.370; p < 0.001). Conversely, ETI in cardiac arrest (vs. non-cardiac arrest; aOR 0.677; 95% CI 0.538–0.852; p < 0.001) and the presence of a difficult airway (vs. non-difficult airway; aOR 0.324; 95% CI 0.254–0.412; p < 0.001) were significantly associated with decreased FAS.

**Table 3:** Univariable and multivariable analyses to identify the association between FAS and cumulative ETI cases.

	Univariable			Multivariable		
	OR	95% CI	*p*-value	OR	95% CI	*p*-value
**Cumulative number of ETI cases per trainee**	1.007	1.007–1.010	< 0.001	1.010	1.006–1.013	< 0.001
**Patient age**	1.004	0.998–1.011	0.202			
**Patient sex, male**	0.879	0.706–1.096	0.252			
**ETI device, VL (vs. DL)**	1.748	1.408–2.171	< 0.001	1.890	1.506–2.370	< 0.001
**BMI**						
Normal weight	Reference					
Underweight	0.997	0.703–1.414	0.986			
Preobese	0.868	0.675–1.116	0.270			
Obese	0.742	0.432–1.275	0.279			
**ETI indication, CA (vs. non-CA)**	0.817	0.659–1.012	0.064	0.677	0.538–0.852	< 0.001
**Anticipated difficult airway (vs. non-difficult airway)**	0.402	0.322–0.503	< 0.001	0.324	0.254–0.412	< 0.001

Generalized linear mixed models with random intercept using the logit link function were used to evaluate the association between FAS and cumulative ETI experience.

*FAS* first attempt success, *ETI* endotracheal intubation, *OR* odds ratio, *CI* confidence interval, *VL* video laryngoscope, *DL* direct laryngoscope, *BMI* body mass index, *CA* cardiac arrest.

### Post-hoc power analysis

Power analyses were performed using a coefficient value that was less than the estimated regression coefficient to explore whether the sample size used in our retrospective analysis was sufficient or not^14^. The power to detect a regression coefficient (e.g., 0.005) smaller than the estimated regression coefficient (= 0.006586) of the cumulative number of ETI cases in the prediction model of FAS was calculated over a range of sample sizes using 1000 Monte Carlo simulations (see Supplementary Fig. S3). The range of possible values for the x-axis, representing the cumulative number of ETI cases, ranged from 50 (n = 1006) to 204 (n = 2077). The power of a minimum cumulative number of ETI cases of 119, which was necessary to achieve an FAS probability of 85%, was 83%.

## Discussion

Several studies have been performed to determine the ETI learning curve, with varying results, presumably owing to the differences in the statistical methodologies, expertise of the study subjects, devices used, study setting, and definition of proficiency. In this study, we sought to define the learning curve of ETI for EM trainees in the ED. The cumulative number of ETI cases was significantly associated with an increased FAS in the multivariable model. A minimum of 119 ETI cases were required for EM trainees to achieve an FAS probability of ≥ 85% in our study. We believe that our study is clinically relevant. As ETI has the potential to save lives, EM physicians are expected to be skilled in it. Thus, evidence quantifying the number of ETI cases required to achieve a level of proficiency may serve as a better guide for EM trainee education.

The ACGME has deemed ETI an essential key index procedure for the independent practice of EM, mandating that EM residents perform at least 35 ETIs during their training^1^. According to the anesthetic literature, between 51 and 75 ETIs are necessary to obtain an overall success rate of ≥ 90% when utilizing a DL^9^. A previous meta-analysis on the learning curve of ETI employing a DL found that a minimum of 50 ETIs in an elective setting is required to obtain a success rate of ≥ 90% within two ETI attempts^10^. According to a study conducted by Je et al. for EM residents in the ED, a 90% success rate was not achieved despite having performed 114 ETI procedures^2^. In our study, EM trainees were required to perform a minimum of 119 ETI cases to achieve a FAS probability of ≥ 85%. Even if the criteria for proficiency were based on a FAS rate of ≥ 80% or a success rate of ≥ 90% within two attempts, at least 66 and 88 cumulative ETI cases, respectively, would be necessary. Considering evidence from previous literatures as well as our study findings, the 35 ETI cases may not be sufficient for establishing the proficiency of EM trainees. ETI in the ED is typically challenging due to the uncontrolled environment, critically-ill patients with compromised physiology, and frequently encountered difficult ETIs, leading to a variety of life-threatening complications. Thus, additional training for EM trainees might be necessary to be proficient in ETI.

Our study found that managing a substantial number of cases is necessary to develop proficiency in ETI. One possible explanation is that we used an FAS rate of ≥ 85%, which might be stricter compared to the criteria in other studies, as a proxy for ETI proficiency. The more difficult it was to achieve this level of expertise, the more experience was necessary. Second, it has been well established that incidences of a difficult or failed ETI are much higher in emergent settings than in elective settings. Patients with difficult airway characteristics and those who had suffered a cardiac arrest, both of which might have a detrimental effect on the FAS rate, accounted for 25.5% and 37.2% of the patients in our study, respectively. Kim et al. reported that 137 and 243 ETI cases were necessary to achieve a 90% success rate without complications within 60 and 30 s, respectively, during cardiopulmonary resuscitation^3^. Finally, the approach used to collect the data was different from that used in other studies. Our ETI attempts and successes were captured in real time by dedicated observers, ensuring objectivity of the data^12^. Studies that rely on recalled or self-reported data may be prone to bias in ETI attempt numbers and success rates.

In this study, we used a FAS rate of ≥ 85% as an indicator of proficiency. To date, there has been no consensus over the definition of ETI proficiency. Previous research evaluating the learning curve for ETI has employed various definitions of competency, such as a success rate of at least 90% in less than 30 s or a success rate of ≥ 90% within two attempts^4,10^. Current practice now focuses, as a priority, on successful ETI on the first attempt, as several studies have shown that increased ETI attempts are associated with a higher risk of adverse events (AEs). According to Sackles et al.’s study that analyzed 1828 ETI in the ED, the incidence of AEs was 14.2% in patients with FAS, but it increased to 47.2% and then to 63.6% as the number of attempts increased, reaching 70.6% in cases with more than four attempts^15^. In a study involving non-traumatic out-of-hospital cardiac arrest patients, Murphy et al. also showed that the likelihood of achieving a favorable neurologic outcome decreased with each successive attempt: 11% with one attempt, 4% with two attempts, 3% with three attempts, and 2% with four or more attempts^16^. These data suggest that the goal of emergency ETI should be to maximize the FAS, emphasizing the importance of future educational initiatives aimed at increasing the FAS rate. Although FAS is essential for patient safety, there are no universally accepted specific values for measuring proficiency. Several studies conducted in the ED have indicated that the FAS rate is often around 80–90%^11,17–22^. Park et al. conducted a systematic meta-analysis on data collected from 42,081 patients intubated in the EDs of 83 institutions located across ten counties^11^. This data showed that the FAS rate in the ED was 84.1% (95% CI 80.1–87.4]). Based on the findings of previous studies, as well as the importance of the FAS for patient safety, it may be reasonable to establish a FAS target of ≥ 85% for EM trainees; hence, this criterion was selected as the primary outcome of this study.

Simulation-based training for ETI enables guided experiences in safe environments, thereby possibly facilitating the attainment of the level of proficiency required to safely operate in an actual clinical setting. Despite the fact that studies on the effectiveness of simulation-based training have yielded varying degrees of success based on study methodology, subjects, and outcomes of interest, several studies have shown favorable findings for the efficacy of simulation-based training. Mosier et al. implemented a 3-year training program including an intensive simulation-based curriculum that emphasized the recognition and preparation for potentially difficult ETI cases and procedural skills^23^. This curriculum increased the FAS rate from 74 to 82% (P = 0.006) and reduced the occurrence of complications, such as desaturation rates, from 26 to 17% (P = 0.002). Our study did not assess the effect of simulation-based training. However, because EM physicians need to manage airways in a variety of scenarios, continuous and comprehensive simulation-based training that includes other components of airway management, such as preparation and patient evaluation, in addition to training to acquire the technical skills of ETI, may be beneficial in reducing the number of ETI required to achieve proficiency in ETI.

There are several limitations in our study, which should be noted when interpreting the results. First, because this study was conducted in a single academic ED, its findings may not be generalizable to other settings. In particular, because the majority of ETIs were performed using conventional DL and C-MAC VL, there may be limits to extrapolating our findings when alternative devices are employed. Second, this was an observational retrospective study conducted using an institutional registry. As a result, the number of ETIs could not be controlled for each trainee, resulting in a wide range of ETI cases per trainee. Additionally, selection bias may exist in the choice of airway device based on the intubator’s preference or the presence of difficult airway characteristics. It was challenging to design a study in which airway equipment was randomly assigned for research purposes in clinical practice. It is reasonable for the intubator to choose the device that most effectively secures the airway in each scenario. Third, we analyzed only ETIs conducted in the ED, but not those performed during rotations in other departments, such as the intensive care unit. In addition, we did not consider prior ETI experiences before starting the residency program. However, this is likely to have minimal effect because the majority of trainees did not have prior ETI experience. Taken together, the number of ETI cases for trainees in this study was equivalent to or less than the actual number of ETIs performed. Thus, our findings may have been influenced by the uncounted ETI cases. Fourth, the duration of the ETI attempt, another relevant indicator for determining proficiencies, could not be presented because the airway registry lack information on the attempt duration for some patients. Fifth, repeated laryngoscope insertion may increase the incidence of airway obstruction and reduce the success rate of subsequent attempts. Consequently, following the failure of the initial ETI, successive intubations would have been performed under more challenging conditions by other EM trainee. However, only a small number of patients underwent ETI by two or more EM trainees; thus, the impact of this limitation might not be significant. Sixth, the evaluation of difficult airway characteristics was highly susceptible to subjectivity and interobserver variability, especially the assessment of “external appearance,” which includes ambiguous phrases like “short neck” and “small mandible”. Seventh, obesity, a well-known risk factor for ETI failure, was not statistically significant for FAS in our study. This finding may be attributed to a decrease in statistical power resulting from our study's relatively small sample size of obese patients. Finally, we documented all ETI-related complications that occurred during the procedure. However, these complications could not be linked to the individual intubator.

In conclusion, the cumulative number of ETI cases was associated with an increased FAS rate. To achieve an FAS rate of ≥ 85%, at least 119 ETI cases were required for EM trainees in the ED. These findings may have implications for the minimum training number of ETI cases required for EM training.

## Supplementary Information

Supplementary Information.

## Data Availability

The datasets used in this work are available upon reasonable request from the corresponding author and are not publicly available.
