# Supplementary material for: Defining the learning curve for endotracheal intubation in the emergency department
Source: Sci Rep. 2022 Sep 1;12:14903. doi: 10.1038/s41598-022-19337-8 (PMC9437073; doi:10.1038/s41598-022-19337-8)
Supplement: Supplementary file 1 — Supplementary Information. [file 41598_2022_19337_MOESM1_ESM.docx]

**Supplementary Material**

**Supplementary Table S1. Combination of devices used for the first and second ETI attempts and the success rate of the second attempt**

| **First attempt** | **Second attempts** | **Success rate at the second attempt (n=228)** |
| --- | --- | --- |
| DL | DL | 57.4%, (n = 35/61) |
| DL | C-MAC VL | 71.4%, (n = 15/21) |
| DL | PAS | 22.2%, (n = 2/9) |
| C-MAC VL | DL | 90.9%, (n = 10/11) |
| C-MAC VL | C-MAC VL | 63.7%, (n = 65/102) |
| C-MAC VL | PAS | 50.0%, (n = 1/2) |
| PAS | DL | 72.7%, (n = 8/11) |
| PAS | C-MAC VL | 75.0%, (n = 3/4) |
| PAS | PAS | 42.9%, (n = 3/7) |
|  |  | 62.3%, (n = 142/228) |

ETI, endotracheal intubation; DL, direct laryngoscope; VL, video laryngoscope; PAS, Pentax Airwayscope

**Supplementary Table S2. Univariable and multivariable analyses to identify the association between first attempt success and training duration.**

|  | **Univariable** | | | **Multivariable** | | |
| --- | --- | --- | --- | --- | --- | --- |
|  | **OR** | **95% CI** | ***p*-value** | **AOR** | **95% CI** | ***p*-value** |
| **Duration of training** |  |  |  |  |  |  |
| First-year | Reference |  |  | Reference |  |  |
| Second-year | 1.762 | 1.335-2.325 | <0.001 | 2.076 | 1.548-2.784 | <0.001 |
| Third-year | 2.140 | 1.592-2.876 | <0.001 | 2.670 | 1.954-3.647 | <0.001 |
| Fourth-year | 2.675 | 1.738-4.116 | <0.001 | 3.609 | 2.308-5.643 | <0.001 |
| Fellow | 1.579 | 0.682-3.656 | 0.286 | 2.860 | 1.231-7.116 | 0.015 |
| **ETI device, VL (vs. DL)** | 1.748 | 1.408-2.171 | <0.001 | 1.917 | 1.528-2.406 | <0.001 |
| **ETI indication, CA (vs. non-CA)** | 0.817 | 0.659-1.012 | 0.064 | 0.649 | 0.514-0.818 | <0.001 |
| **Anticipated difficult airway (vs. non-difficult airway)** | 0.402 | 0.322-0.503 | <0.001 | 0.331 | 0.260-0.421 | <.001 |

OR, odds ratio; AOR, adjusted odds ratio; CI, confidence interval; ETI, endotracheal intubation; VL, video laryngoscope; DL, direct laryngoscope; CA, cardiac arrest.

To evaluate the distinct influence of trainee level on FAS rate, additional analyses were carried out. However, because there was a correlation between the cumulative number of ETI cases and training duration, a multivariable analysis was conducted by substituting the training duration for the cumulative number of ETI cases. Even when the training duration was considered in the multivariable analysis, there was no change in either the overall significance or odds ratio of other variables.

**Supplementary Table S3. Multivariable analyses to identify the association between first attempt success and cumulative number of ETI cases according to each component of difficult airway characteristics.**

|  | **OR** | **95% CI** | **P-value** | **AOR** | **95% CI** | ***p*-value** |
| --- | --- | --- | --- | --- | --- | --- |
| **Model 1: External appearance** | | | | | | |
| Cumulative Number of ETI cases |  |  |  | 1.010 | 1.007-1.013 | <0.001 |
| ETI device, VL (vs. DL) |  |  |  | 1.866 | 1.488-2.340 | <0.001 |
| ETI indication, CA (vs. non-CA) |  |  |  | 0.691 | 0.550-0.868 | 0.002 |
| External appearance | 0.388 | 0.307-0.491 | <0.001 | 0.310 | 0.241-0.399 | <0.001 |
| **Model2: Mallampati score 3 or higher** | | | | | | |
| Cumulative Number of ETI cases |  |  |  | 1.008 | 1.004-1.011 | <0.001 |
| ETI device, VL (vs. DL) |  |  |  | 1.812 | 1.453-2.259 | <0.001 |
| ETI indication, CA (vs. non-CA) |  |  |  | 0.802 | 0.643-1.001 | 0.051 |
| Mallampati score 3 or higher | 0.678 | 0.344-1.340 | 0.264 | 0.580 | 0.129-1.172 | 0.129 |
| **Model3: Airway obstruction** | | | | | | |
| Cumulative Number of ETI cases |  |  |  | 1.008 | 1.005-1.012 | <0.001 |
| ETI device, VL (vs. DL) |  |  |  | 1.865 | 1.490-2.334 | <0.001 |
| ETI indication, CA (vs. non-CA) |  |  |  | 0.740 | 0.591-0.926 | 0.008 |
| Airway obstruction | 0.203 | 0.123-0.334 | <0.001 | 0.152 | 0.090-0.256 | <0.001 |
| **Model 4. Cervical immobilization** | | | | | | |
| Cumulative Number of ETI cases |  |  |  | 1.007 | 1.004-1.010 | <0.001 |
| ETI device, VL (vs. DL) |  |  |  | 1.864 | 1.493-2.326 | <0.001 |
| ETI indication, CA (vs. non-CA) |  |  |  | 0.816 | 0.655-1.016 | 0.070 |
| Cervical immobilization | 0.495 | 0.324-0.757 | <0.001 | 0.466 | 0.301-0.720 | 0.001 |
| **Model 5. Limited mouth opening** | | | | | | |
| Cumulative Number of ETI cases |  |  |  | 1.008 | 1.005-1.011 | <0.001 |
| ETI device, VL (vs. DL) |  |  |  | 1.833 | 1.468-2.288 | <0.001 |
| ETI indication, CA (vs. non-CA) |  |  |  | 0.787 | 0.631-0.982 | 0.034 |
| Limited mouth opening | 0.332 | 0.196-0.562 | <0.001 | 0.254 | 0.146-0.443 | <0.001 |

AOR, adjusted odds ratio; CI, confidence interval; ETI, endotracheal intubation; VL, video laryngoscopy; DL, direct laryngoscope; CA, cardiac arrest.

Given that some difficult airway characteristics were related to each other in our data and that some patients had two or more characteristics, the model was analyzed separately for each characteristic.

**Supplementary Table S4. Univariable analyses to identify the association between first attempt success and body mass index.**

| **BMI** | **OR** | **95% CI** | ***p*-value** |
| --- | --- | --- | --- |
| Normal weight (18.5–24.9 kg/m^2^) | Reference |  |  |
| Underweight (< 18.5 kg/m^2^) | 0.997 | 0.703 - 1.414 | 0.988 |
| Preobese (25.0–29.9 kg/m^2^) | 0.868 | 0.675 - 1.116 | 0.270 |
| Obesity (30.0–39.9 kg/m^2^) | 0.785 | 0.445 - 1.384 | 0.403 |
| Morbidly obesity (≥40.0 kg/m^2^) | 0.378 | 0.062 - 2.293 | 0.290 |

BMI, body mass index; OR, odds ratio; CI, confidence interval

We conducted a univariable analysis by classifying obese patients as either “obese” (BMI: 30.0–39.9 kg/m^2^) or “morbidly obese” (BMI ≥ 40 kg/m^2^) to identify whether the lack of statistical significance for the effect of obesity on first attempt success was due to patients included in the lower end of the BMI criterion for obesity.

**Supplementary Figure S1. The relationship between the cumulative number of ETI cases and predicted probability of first attempt success depending on the situation. The probability of first attempt success was calculated using the regression coefficient of multiple regression analysis based on the intubating device, indication, and presence of difficult airway characteristics.**FAS, first attempt success; ETI, endotracheal intubation

1. Direct laryngoscope, non-cardiac arrest, and absence of difficult airway characteristics


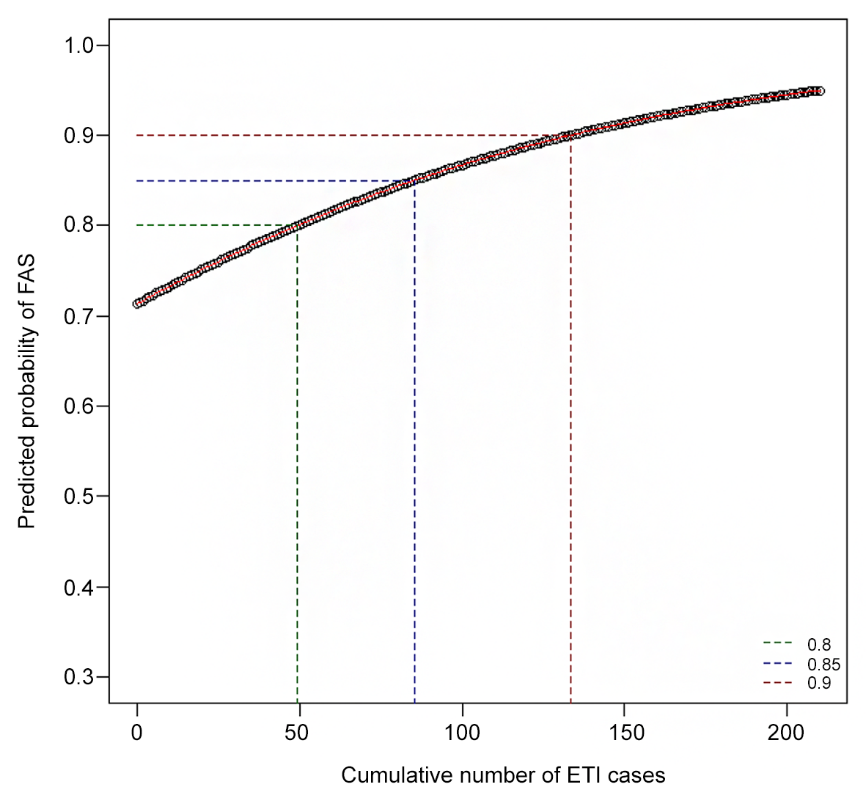


| **Predicted probability of FAS** | **Cumulative cases** |
| --- | --- |
| 0.8 | 49.4 |
| 0.85 | 85.5 |
| 0.9 | 133.5 |

1. Direct laryngoscope, non-cardiac arrest, and presence of difficult airway characteristics


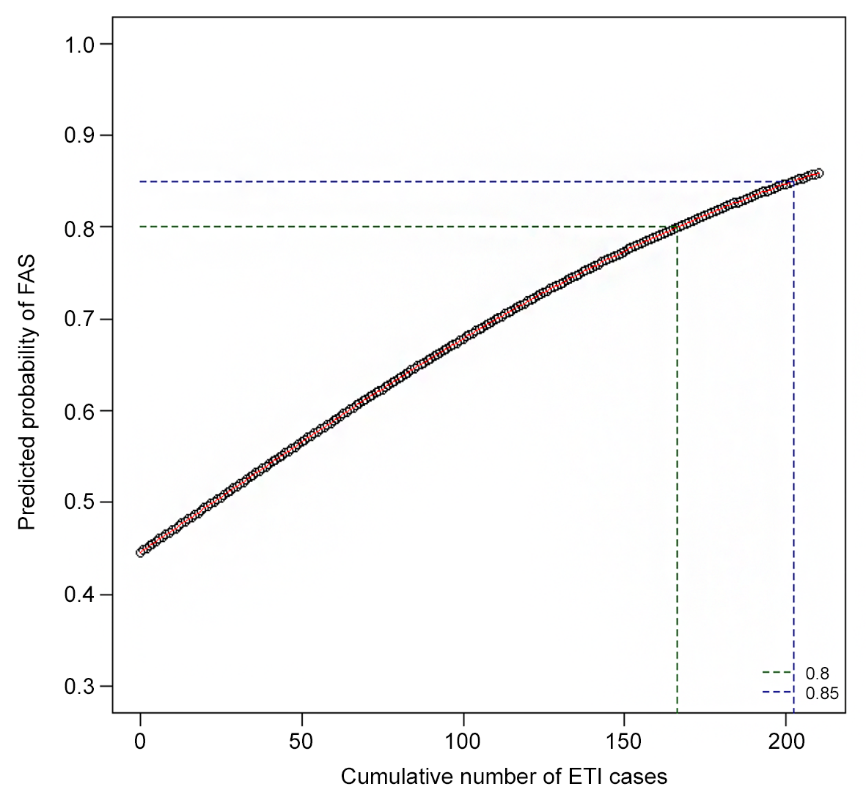


| **Predicted probability of FAS** | **Cumulative cases** |
| --- | --- |
| 0.8 | 166.4 |
| 0.85 | 202.5 |
| 0.9 | - |

1. Direct laryngoscope, cardiac arrest, and absence of difficult airway characteristics


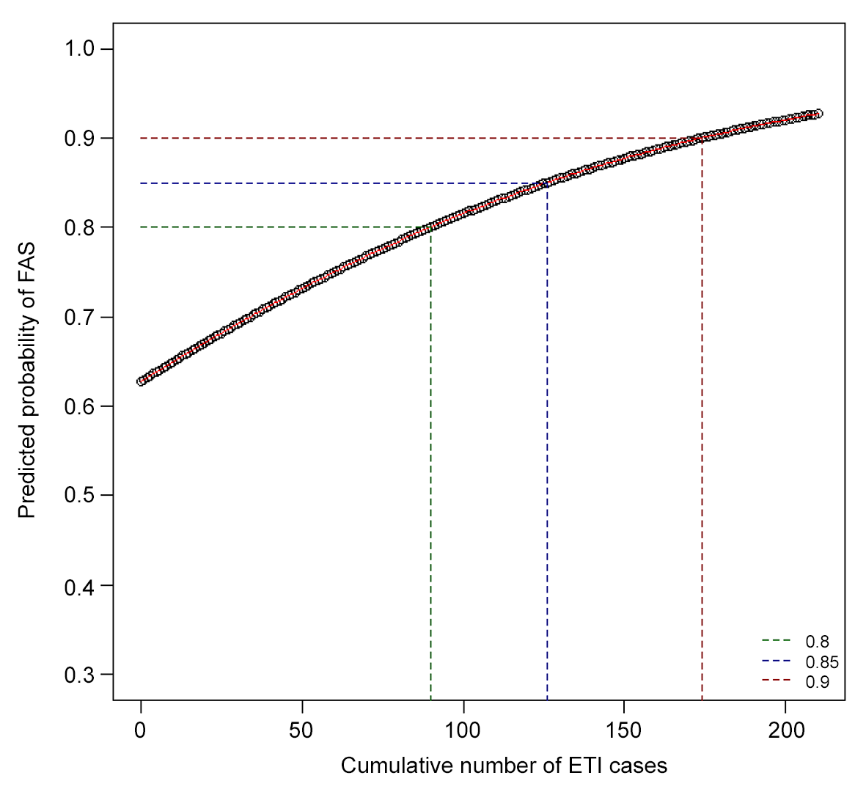


| **Predicted probability of FAS** | **Cumulative cases** |
| --- | --- |
| 0.8 | 89.8 |
| 0.85 | 126.0 |
| 0.9 | 174.0 |

1. Direct laryngoscope, cardiac arrest, and presence of difficult airway characteristics


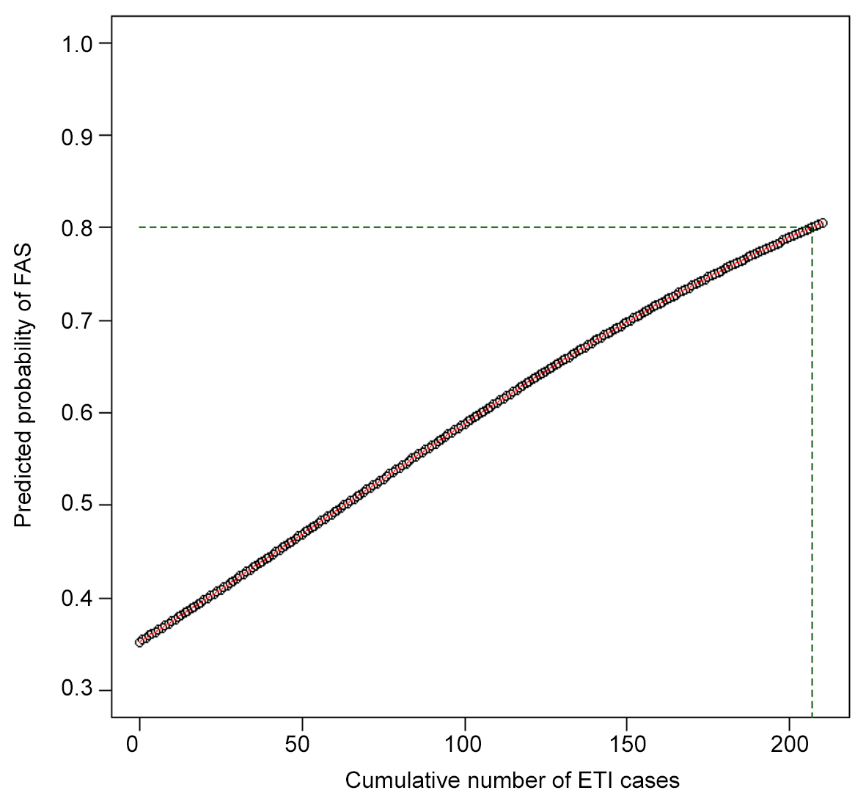


| **Predicted probability of FAS** | **Cumulative cases** |
| --- | --- |
| 0.8 | 206.8 |
| 0.85 | - |
| 0.9 | - |

1. Video laryngoscope, non-Cardiac arrest, and absence of difficult airway characteristics


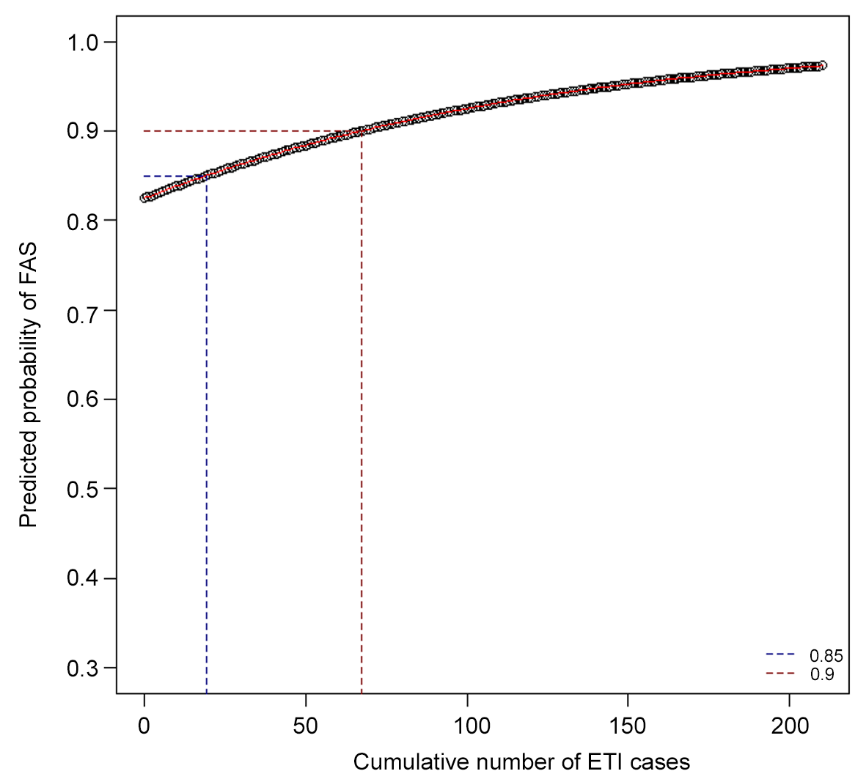


| **Predicted probability of FAS** | **Cumulative cases** |
| --- | --- |
| 0.8 | - |
| 0.85 | 19.5 |
| 0.9 | 67.5 |

1. Video laryngoscope, non-cardiac arrest, and presence of difficult airway characteristics


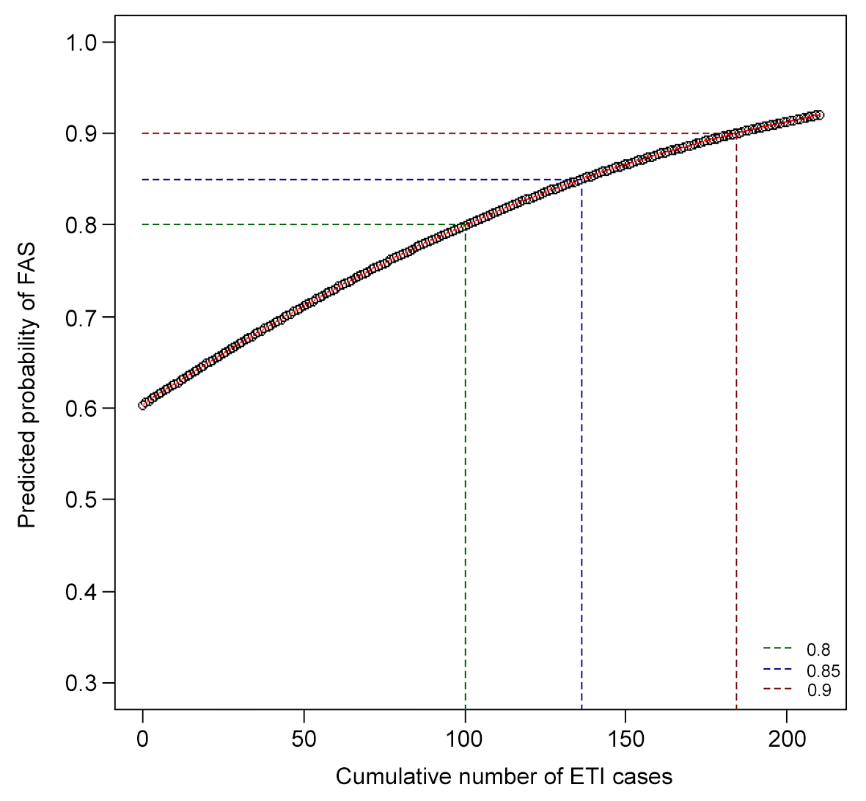


| **Predicted probability of FAS** | **Cumulative cases** |
| --- | --- |
| 0.8 | 100.3 |
| 0.85 | 136.5 |
| 0.9 | 184.5 |

1. Video laryngoscope, cardiac arrest, and absence of difficult airway characteristics


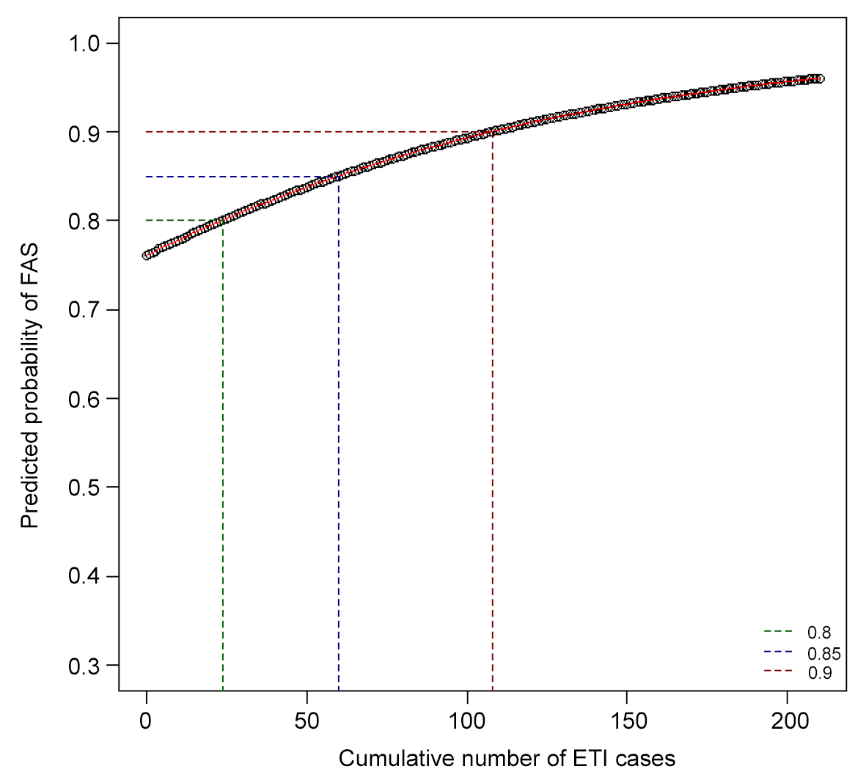


| **Predicted probability of FAS** | **Cumulative cases** |
| --- | --- |
| 0.8 | 23.8 |
| 0.85 | 60.0 |
| 0.9 | 108.0 |

1. Video laryngoscope, cardiac arrest, and presence of difficult airway characteristics


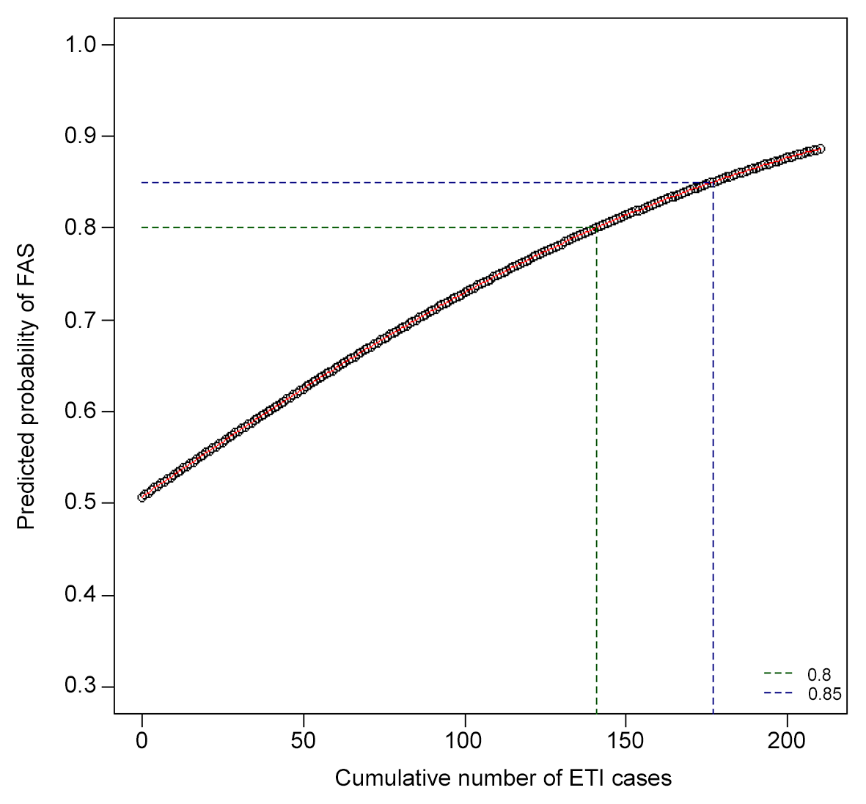


| **Predicted probability of FAS** | **Cumulative cases** |
| --- | --- |
| 0.8 | 140.8 |
| 0.85 | 176.9 |
| 0.9 | - |

**Supplementary Figure S2. The relationship between the cumulative number of cases and the success rate of ETI within two attempts. ETI, endotracheal intubation**


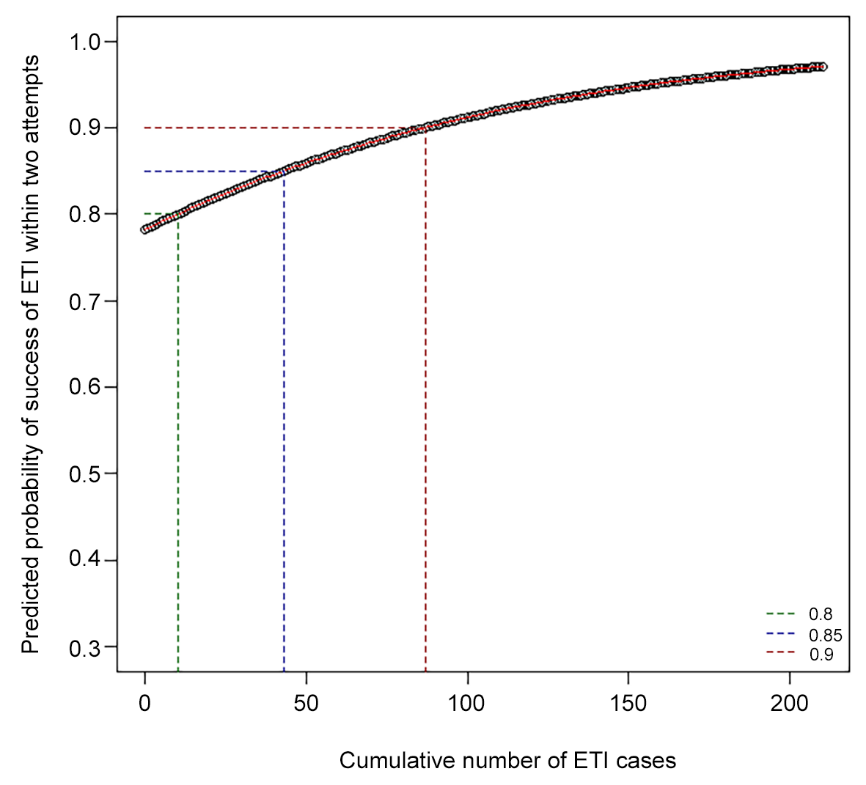


| **Predicted probability of success of ETI within two attempts** | **Cumulative cases** |
| --- | --- |
| 0.8 | 10.5 |
| 0.85 | 43.4 |
| 0.9 | 87.1 |

**Supplementary Figure S3. Monte Carlo simulations with 1000 trials for calculating the power to detect a regression coefficient (e.g., 0.005) smaller than the estimated regression coefficient (=0.006586) of the cumulative number of ETI cases in the prediction model of first attempt success.** ETI, endotracheal intubation


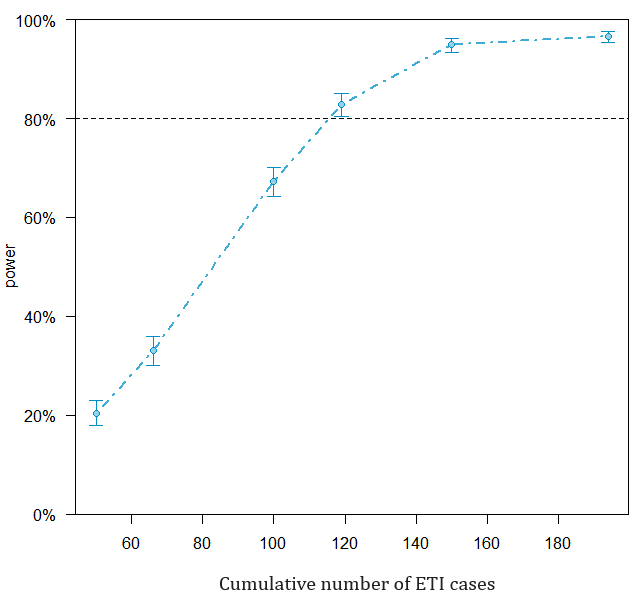


| Cumulative Cases | N | Power |
| --- | --- | --- |
| 50 | 1,006 | 0.204 |
| 66 | 1,305 | 0.330 |
| 100 | 1,720 | 0.673 |
| 119 | **1,862** | **0.829** |
| 150 | 2,017 | 0.950 |
| 189 | 2,067 | 0.967 |
| 204 | 2,077 | 0.983 |
